# Optimization of an HTRF Assay for the Detection of Soluble Mutant Huntingtin in Human Buffy Coats: A Potential Biomarker in Blood for Huntington Disease

**DOI:** 10.1371/currents.RRN1205

**Published:** 2010-12-29

**Authors:** Miriam Moscovitch-Lopatin, Andreas Weiss, Herminia Diana Rosas, James Ritch, Gheorghe Doros, Kimberly B. Kegel, Marian Difiglia, Rainer Kuhn, Graeme Bilbe, Paolo Paganetti, Steven Hersch

**Affiliations:** ^*^Massachusetts General Hospital/Harvard Medical School, Boston, Massachusetts; ^†^Novartis Institutes for Biomedical Research, Neuroscience; ^¶^Boston University; ^#^Massachusetts General Hospital/Harvard Medical School; ^**^Massachusetts General Hospital; ^††^Neuroscience Research, Head Neurodegeneration at Novartis, Basel, Switzerland; ^‡‡^Novartis Institutes for Biomedical Research and ^§§^AC Immune SA

## Abstract

A means for measuring levels of soluble huntingtin proteins in clinical samples is essential for assessing the biological effects of potential mutant huntingtin (mtHtt) modifying treatments being developed for Huntington’s disease (HD). We have optimized a previously described cell-based Homogeneous Time Resolved Fluorescence method that can measure soluble mtHtt and its ratio to the total Htt (tHtt) in blood buffy coats [1]. The results of the optimization and assay qualification indicate the assay to be specific for mtHtt in HD compared to Control subjects, highly sensitive, and technically and biologically reproducible. We therefore generated a Good Laboratory Practice Standard Operating Procedure which we validated, using 30 HD and 8 control buffy coat samples in which significant differences in mtHtt levels were found. We intend to deploy the assay to evaluate sample sets from observational and therapeutic studies enrolling HD subjects to further validate soluble mtHtt measurement by HTRF as a biomarker for HD and to explore its potential uses.

**Introduction**

The proximate cause of neuronal dysfunction and death in Huntington Disease (HD) is the presence of soluble mutant huntingtin (_mt_Htt) protein or its cleavage products  [1][[2] [3]. Consequently, soluble _mt_Htt is the most salient target for disease modifying therapies and measurement of soluble _mt_Htt is essential for evaluating the success of therapies intending to affect _mt_Htt levels. Using a cell based Homogeneous Time-Resolved Foerster Resonance Energy Transfer (HtrFRET) assay or HTRF, Weiss et al [1]have shown that _mt_Htt was specifically detectable in human brain and blood cell sub-populations, as well as in tissues and blood from HD mouse models, in correlation with the disease. Furthermore, using properties of Lumi4-Terbium cryptate (Tb) Weiss et al [4] have developed a triplex assay, in which _mt_Htt, as well as an epitope distant from the Htt N-terminus, hence an indication of total huntingtin (_t_Htt), are measured concomitantly in the same sample.

This assay, applied to peripheral blood samples, hence easily accessible, from HD patients, could serve as a biomarker for assessing whether systemic therapies affect levels of _mt_Htt. We chose buffy coat (BC) as a blood subpopulation showing measurable Htt levels (Weiss et al [5] and that is also relatively easy to quantify and work with. To provide the necessary consistency, reliability, and quality control essential for a clinical diagnostic bioassay, we have optimized and qualified the assay for BC samples and developed Standard Operating Procedures (SOPs), so that the assay can be conducted under Good Laboratory Practice (GLP) conditions and in compliance with Federal Drug Administration (FDA) requirements. We first optimized the assay to measure soluble _t_Htt and _mt_Htt in human brain lysates from control and HD subjects. Parameters providing the highest specificity and sensitivity for _mt_Htt were selected and the assay was used to test sets of unblinded human BC samples from HD and control subjects, to qualify/ validate the assay in compliance with GLP. Following the assay qualification, a formal SOP consistent with FDA requirements for a potential diagnostic assay was issued. Using this SOP, we have started to test clinical samples to assess relative blood levels of _mt/t_Htt using blood samples from active single and multi-center clinical research studies. Our results using this SOP confirm that the assay is sensitive, linear within the dynamic range and reliable and we are deploying it as a GLP assay to explore its potential as a peripheral biomarker for HD.

**Material and Methods**

**Antibodies: **The monoclonal antibodies used in this cell-based HTRF assay are specific for selected epitopes on the Htt molecule. The antibodies include: 2B7 [5],which binds to the first 17 amino acids (aa) of normal and _mt_Htt; MW1 [6],which is specific for expanded polyglutamine sequences (polyQ) and binds to _mt_Htt, but not to normal Htt; and 2166 (Millipore Corp, Cat# MAB2166 [7], which binds to a Htt epitope starting at aa 444, and recognizes both normal and _mt_Htt. 2B7 was conjugated to Tb (Cisbio), to serve as the donor for HTRF, while MW1 was bound to AlexaFluor 488 (AF488, Alexa Fluor 488, Invitrogen, Cat#A20181) and 2166 was conjugated to d2 (Cisbio) to serve as acceptors and to enable multiplexing between the donor and the two acceptors. Additional antibody pairs were also evaluated (not shown), however the antibodies mentioned above showed the largest HTRF signals and discrimination between the controls and HD and were therefore selected for the optimization study.

**Human Samples: **Human blood samples were collected at the MGH Huntington’s Disease Center by standard phlebotomy from consenting HD and control subjects participating in the Partners IRB approved REVEAL-HD study. Genotyping was confirmed by the MacDonald laboratory (Molecular Neurogenetics Unit Genotyping Resource) at MGH. Twenty-four of these samples were from 12 individuals in whom venipuncture was repeated within six weeks. The peripheral blood samples, collected into EDTA tubes, were centrifuged at 4°C for 10 minutes (min) at 1000 x g. BC fractions were isolated and centrifuged again for 20 min at 4°C at 15800 x g, then stored at -80°C until their analysis. The BC pellets were pre-diluted 1:2 in “lysis buffer” and an additional two serial two-fold dilutions were performed.

**Brain Tissue Samples: **Postmortem frontal cortex samples from HD and control patients obtained from the Brain Bank of the Alzheimer Disease Research Center (ADRC) at MGH were homogenized in 10x volume “lysis buffer” as above. Stocks of lysates were prepared, their protein concentrations determined and the stocks aliquoted and stored at -80^°^C for use as quality control (QC) samples and for assay development and troubleshooting purposes.

**HTRF Assay** **: **Our optimization process examined plate formats; the performance of various plate readers and filters, reaction volumes, buffer compositions, cell lysate and antibody concentrations, quality control and normalization approaches, and reaction incubation times. Optimal conditions utilized white, 384 well/plate (Perkin Elmer, Cat# 6008280), and a total volume of 15 µL/well. The cell lysates at 12.5 µL/well were derived from a 1:1 dilution of the BC samples and the two subsequent two fold dilutions, or from 9 µg/well of each control and HD brain lysate diluted in the lysis buffer. The fluorophore-conjugated antibodies were in a 2.5 µL/well reaction buffer, that included a final dilution of 1.25ng/well 2B7-Tb cryptate-labeled antibody and 2.35ng/well MW1-AF (different lot than previously mentioned) and 13ng/well 2166-d2-labeled antibodies in 50 mM NaH_2_PO_4_ (NaH_2_PO_4_ –H_2_O, Sigma Aldrich, S9638), 200 mM KF (Sigma-Aldrich, Cat# 402931-100G), 0.1% BSA (Sigma, Cat# A-7906) and 0.05% Tween 20 (Fisher, Cat# BP337-500). The concentrations of conjugated-antibodies are lot dependent, therefore their concentration is adjusted with each new lot to yield similar ratio of _mt/t_Htt of HD/control human brain lysates. The plates were spun for 3 min at 500 x g then incubated in the dark on an orbital shaker at 4°C for 2 hours (hr). The HTRF signals were read on a VICTORX5 plate reader (Perkin Elmer). The emission from Tb was captured at 615 nm, following optimization and we term the values, for simplicity, maximum (Max) signal. The ratio of signals at the 665/615 nm and 510/615 nm, respectively, represents a specific, artifact corrected determination of the two antibody pairs: 2166-d2/2B7-Tb and MW1-AF/2B7-Tb, simultaneously bound to Htt and reveal information about_t_Htt/Max and _mt_Htt/Max signal, respectively.

**dsDNA Content** was measured in all the wells (including the dilutions of each sample) of the assay following the reading of the HTRF signals using PicoGreen (Quant-iT PicoGreen dsDNA Quantitation Kit, Invitrogen, Cat# P11496). A standard curve of dsDNA was generated on every plate, to make sure that the samples measured are within the dynamic range of the standard curve. The plate was read at an excitation of 485 nm and an emission of 535 nm.

The **protein concentrations** of the human brain lysates and BC samples were determined using DC Bio-Rad, Protein Assay Reagents, per the kit instructions.

**Purified 548Q25, 548Q46 and 548Q72 Htt fragments: **Purification of recombinant huntingtin protein fragments was performed as previously described (Weiss et al [[5] [4]). We thawed the cryo-stored purified Htt fragments at room temperature, prior to spiking them into the control buffy coat sample at 1:1 ratio, yielding Htt fragments concentration of 300-0 ng/mL and a 1:4 final dilution of the control BC used. The HTRF assay of the spiked samples and controls proceeded as described above.

**Results**

Tb has high stability and long-lived fluorescence emission over a wide spectrum (480-720 nm) following its excitation at 340 nm. Its emission signal is captured at 615 nm-maximum (Max) signal. The energy transferred from the Tb excites the two acceptors: d2, with the peak emission at 665 nm (red) and AF488 (AF), with its peak emission at 510 nm (green). These filters were fine tuned as a result of optimization of the initial assay (Weiss et al [5]). Time resolved detection based on Tb characteristics eliminates the background fluorescence, resulting in a more specific signal. The interaction between the fluorophores reflects the proximate binding of antibodies that are conjugated to their specific epitopes on the Htt molecule (Weiss et al [4]) in this triplex assay. The antibodies we used in the HTRF assay, their conjugated fluorophores and their peak emissions and the antibodies’ epitopes on the Htt molecule are represented schematically in Figure (Fig.) 1:

**HTRF Assay Schematic Summary**

**Figure:**
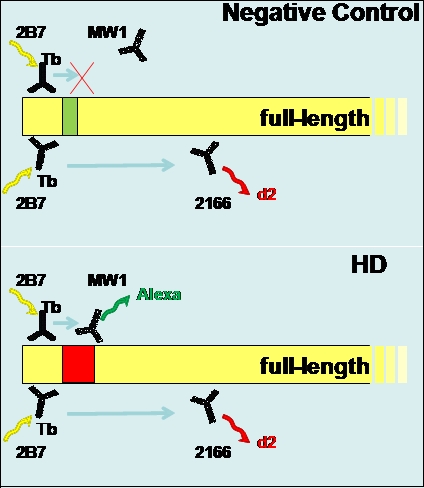
**Fig. 1 HTRF Assay Summary: Schematic representation of the antibodies used in the triplex assay/each well: 2B7, MW1 and 2166, their conjugated fluorophores: Tb, AF488 (Alexa) and d2, respectively, their respective peak emissions:  615 nm, 510 nm and 665 nm, and the antibodies’ binding epitopes on the control and HD Htt molecule, as used in this HTRF assay. In summary:  2B7-Tb-615nm; 2166-d2-665nm, MW1-AF488-510nm.
**

To qualify the HTRF assay and validate soluble _mt_Htt as a potential clinical biomarker, we developed and optimized a protocol based on the Weiss et al assay [5], following GLP, to insure: standardization, reproducibility, clinical utility, the potential for regulatory acceptance, and to provide a basis for future modifications or variations. The immediate technical goal of the optimization process was to develop a protocol that provides the strongest detection of _mt_Htt (MW1/2B7) and _mt/t_Htt (MW1/2166) HTRF signal ratios, using human brain lysates as QCs. Several of the parameters evaluated experimentally for the clinical assay optimization are listed below.

**Plate Format and Plate Color: **We determined that 384 well plates gave a stronger specific signal compared to 96 well plates (results not shown), which is related to the optical interaction of the light, the fluorophores and the height of the reaction fluid (Cisbio, technical information). We compared black and white 384 well plates and evaluated the impact on the relative HTRF signals. We had hypothesized that black plates would reduce the fluorescent background signal better than white plates, however white 384 well plates showed consistently higher HTRF signals compared to black plates. This suggests that the time-resolution fluorescence conferred by the Tb, reduces the background sufficiently, irrespective of plate color and therefore white plates were used from that point forward.

**Reaction Volume and Intra-assay Reproducibility**: We compared (Fig. 2) the effects of different reaction volumes in the triplex HTRF using: 12 µL/well versus 15 µL/well in HD compared to control samples. Our results, represented as average (AVE) of HTRF raw signals ± standard deviation (STDEV) of the triplicates, indicate that at 15 µL/well there is better intra-assay reproducibility than at 12 µL/well for all the antibodies/fluorophores combinations: 2166-d2, 2B7-Tb and MW1-AF488, measured in the same well. The smaller coefficients of variation (CV) between the replicate wells increase confidence in the accuracy of the measurement and the signal seems more specific showing a higher signal with MW1-AF488 for HD compared to controls. Therefore, going forward we used 15 µL/well as the reaction volume.

**Figure:**
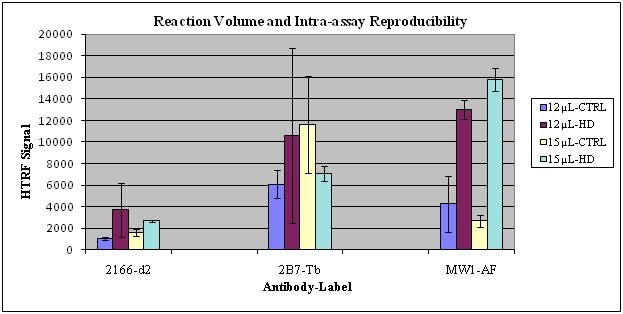
**Fig. 2: Reaction Volume and Intra-assay Reproducibility **Control (CTRL) and HD brain lysates were assayed comparing 12 µL/well versus 15 µL/well total volume reaction in the triplex HTRF. While concentrations of the reagents were as described in the Material and Methods, the incubation time in this experiment was only 1 hr at 4^°^C. Averages (AVE) of HTRF raw signals of triplicates ± standard deviation (STDEV) are shown.

**Reaction Media (KF concentration): **We compared (Table 1) concentrations of 83 mM KF to 200 mM KF in the reaction media (concentrations in use by different labs) and we found that 200 mM KF gave higher signal ratios, due to reduced non-specific binding in the control brain lysates, therefore we adopted 200 mM KF for our reaction media in the HTRF assay.

**Table:** 

**Ratios**	**83 mM KF**		**200 mM KF**	
	**CTRL**	**HD**	**CTRL**	**HD**
**MW1/2B7 (520/615nm)**	**0.7**	**4.3**	**0.4**	**3.3**
**2166/2B7 (665/615nm)**	**0.5**	**0.4**	**0.4**	**0.3**
**MW1/2166 (520/665nm)**	**1.4**	**11.8**	**1.2**	**11.0**
**HD/CTRL-MW1/2B7**	**6.2**		**7.7**	
**HD/CTRL-2166/2B7**	**0.7**		**0.8**	
**HD/CTRL-MW1/2166**	**8.3**		**9.2**	

**Table 1.** **Reaction Media- KF Concentration **Control (CTRL) and HD brain lysates were tested using 384 white microtiter plates with 15 µL/well total volume. The antibodies concentrations were as described in the Material and Methods; the reaction media included 83 mM KF or 200 mM KF.

**HTRF Reaction Time Course**: Evaluation of the time course of the HTRF assay (Fig. 3) revealed an increase in the _mt/t_Htt (MW1-AF/2166-d2) of HD/control HTRF signal peaking at 2-3 hrs, followed by a decrease in this ratio from 9.2 at 3 hrs to 5.0 at 23 hrs, due mostly to an increase with time of the non-specific binding of MW1-AF488 in the CTRL brain. We therefore chose a 2 hr reaction time for the SOP. Stability of the signal within the 2- to 3 hrs incubation interval, indicates that the assay is robust.

**Figure:**
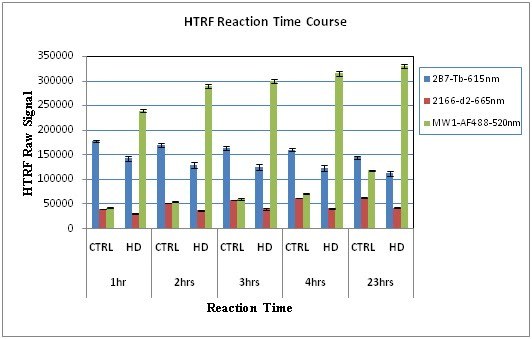
**Fig. 3: Effect of the Reaction Time on Htt Detection by HTRF **The raw HTRF signals’ averages (AVE) of triplicates ± standard deviation (STDEV), measured at 615 nm (2B7-Tb), 665 nm (2166-d2) and 520 nm (note that 520 nm filter was used for MW1-AF488 prior to the final optimization) of CTRL and HD brain lysates were measured in the same plate at 1 hr, 2 hrs, 3 hrs, 4 hrs and at 23 hrs. The assay was performed as described in Material and Methods, with the modifications mentioned. Between measurements, the reaction continued in the plate by mixing on the orbital shaker at 4^°^C. 

**Quality Control:**

**A. Consistency of Signal across the Plate: **Plate based assays can provide inconsistent signals across the plate: top to bottom and/or left to right. Since our goal is to detect small changes in subjects with disease progression or changes due to a _mt_Htt altering therapy it is important to minimize and account for such potential variability. To this end we evaluated 384 well white plates from different manufacturers and we also assessed this parameter when we evaluated the different plate readers by testing brain lysates from control and HD subjects in triplicates at the four corners of the plates. The signal consistency is evaluated as the Z’ defined as:

**Figure:**
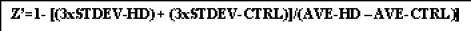

      For HTRF assays a Z’  value ≥0.5 is considered satisfactory. 

      Although minimal, we observed that the left to right variability was lower than top to bottom variability in the plates we determined experimentally to be best for our application. We therefore use only the top 60% of the plate, and the entire plate from left to right. To minimize and control for potential plate related variability we measured Htt in QC samples, control and HD brain lysates, in triplicates on both left and right extremes of each plate when testing clinical samples and calculated the Z’ based on a total of 6 replicates each for control and HD.  A Z≥0.5 was determined as a QC’ acceptance criteria for the assay.

- **B. Signal Normalization: **
- Since the binding to Htt of all the antibodies involved occurs simultaneously in the same cell lysate/well, we can normalize the signal for
- _mt_
- Htt to the signal for
- _t_
- Htt resulting in a ratio-
- _mt/t_
- Htt that identifies each sample. Furthermore, these ratios allow comparisons between different samples to assess the relative levels of
- _mt/t_
- Htt at the time of measurement. Since there is variability in the concentration of cells/mL of blood based on processing variations, sex, age, and other factors we make every attempt to compare the
- _mt/t_
- Htt ratios between samples corresponding to similar dsDNA content and/or protein concentrations, which are valuable QC factors. This is facilitated by measuring dsDNA, using PicoGreen, in all the wells, following the reading of the HTRF signals and by measuring the protein concentration of the lysates and comparing the resulting HTRF ratios at the sample dilutions corresponding to similar dsDNA and protein concentrations.

**Plate Reader**: We evaluated the performance of five different plate readers from three manufacturers and found differences (not shown) of up to 46% in regard to the highest HTRF signal ratios for the HD/control brain lysates and signal consistency across the plate (Z’ values). In addition, cost and technical support played a role in the decision process.  We selected the VICTORX5 (Perkin Elmer) as our plate reader, and the assay qualification/validation and the analyses of the BC and QC samples that followed were performed using it. We started to test the clinical samples following the installation of Security Software and the implementation of Installation Qualification (IQ) and Operational Qualification (OQ) by the VICTOR5X manufacturer, as required by the FDA for a potential diagnostic assay.

**Assay Qualification/Validation Using Clinical Buffy Coat and QC Samples: **Using the optimized protocol and the VICTOR5X we proceeded to confirm the specificity, linearity and sensitivity of the assay. We then proceeded with assay qualification/validation to assess the technical and biological reproducibility of the assay using blood samples such as those intended for clinical use of the assay.

Blood samples from eight individuals including HD subjects and negative controls were divided into three equal sample sets. Each blood sample from each set was processed to obtain the BC and the samples were tested in triplicates along with two sets of QC samples on both the left and right edges of each plate. The three assays were performed over several days, by two operators, using replicate aliquots of the reagents.

**Assay Specificity: **We confirmed the assay’s specificity, using our GLP SOP protocol, as shown in Fig. 4 for human BC samples and the brain QC samples, and demonstrated a significant difference between HD and control subjects in regard to the _mt_Htt and _mt/t_Htt HTRF signal ratios.

**Figure:**
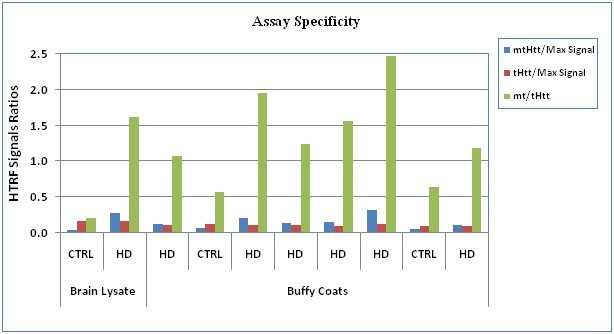
**Fig. 4: Assay Specificity in 2 controls (CTRL)and 6 HD subjects **The HTRF ratios of the MW1-d2/2B7-Tb signals (_mt_Htt/Max), 2166-d2/2B7-Tb signals (_t_Htt/Max), and MW1-d2/2166-d2 signals (_mt/t_Htt) are shown. The assay was performed using the protocol per the SOP, as described in Material and Methods.

**Assay Linearity: **Every BC sample was analyzed at 3 dilutions, corresponding to approximately 1:2, 1:4 and 1:8 final dilutions (Fig. 5). The control and HD brain lysates were used at a constant protein concentration (9 µg/mL) on every plate, a concentration that was determined experimentally (not shown) to be within the linear range for these samples and within the range of values obtained for BC samples in HTRF, dsDNA and protein measurements. The decrease in the signal for the control BC samples correlates most likely to the dilution of the matrix effect (the influence of other cell lysate components). We noticed a linear decrease of signal in HD samples with lower _mt/t_Htt. In samples with moderate to low Htt levels, the signal decreases in a linear fashion with the sample’s dilution, however, in samples with high Htt levels, the signal is usually inhibited at the lowest (1:2) dilution of the lysates, it peaks at the 1:4 dilution and then decreases, resulting in a linear dose response within 1:4 to 1:8 dilutions. The existence of a linear dose response curve in the HD BC samples provides the dynamic range necessary for the relative quantitation, in conjunction with QC criteria (dsDNA and protein concentration mentioned above). The inhibitory effect of concentrated lysates on the _mt/t_Htt HTRF signal is most likely due to excess of antigen (Htt) and/or matrix effect, as is often seen in such situations in ELISA and other immunoassays.

**Figure:**
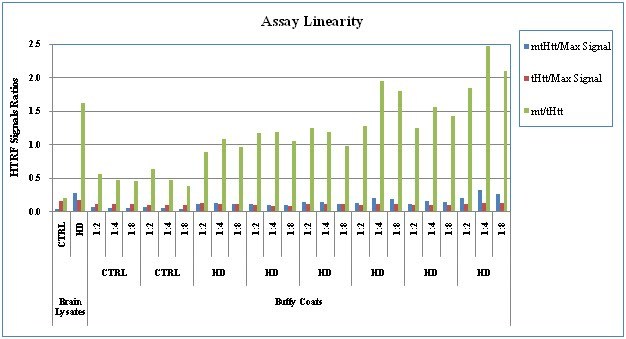
**Fig. 5: Assay Linearity **The linearity of the assay is shown in a representative experiment, performed as described in Material and Methods, where the dilutions performed  for each buffy coat of the 2 control (CTRL) and 6 HD samples and their resulting HTRF ratios are shown.

**Relative Assay Sensitivity**: Spiking of control BC sample with purified full-length _mt_Htt is not useful for absolute quantitation, since Htt will be affected by the aggregation of the mutant and normal protein in our testing conditions. Therefore, this is a semi-quantitative assay, in which we can only evaluate relative signals in HD samples compared to controls. To gain information on the recovery of full-length _mt_Htt and relative sensitivity of the measured _mt_Htt in our assay we spiked control brain lysates with increasing amounts of HD brain lysates to yield ratios as shown in Fig. 6 (ratios of CTRL: HD from 90:10 to 10:90).It is difficult to calculate the exact % recovery and implicit Limit of Quantitation (LoQ) sensitivity, since the control sample contributes as well to the signal, however a signal recovery of approximately 80-100% is apparent. The dynamic range resulting in a linear response occurs within 20-60% HD spiked into the control sample, again emphasizing the linear nature of the assay within this range.

**Figure:**
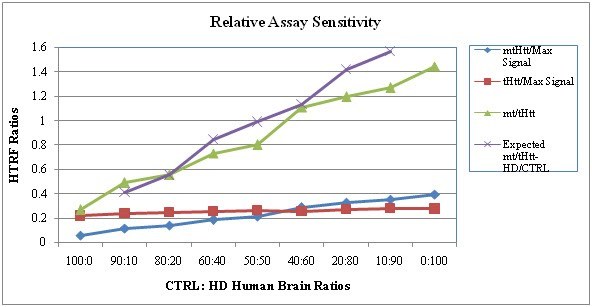
**Fig. 6: Relative Assay Sensitivity: Spike of HD into Control Human Brain Lysates and Signal Recovery **HD brain lysate was spiked into control (CTRL) brain lysate at increasing proportions: 0- 100% to yield a final concentration of  9 µg/well and tested as described in Material and Methods for brain lysates as QC.

To obtain an estimated numeric value for our HTRF measurements and to examine the effects of differing CAG lengths, we spiked purified N-terminus 548 aa Htt fragments: 548Q25, 548Q46, 548Q72, respectively into control BC. The final concentrations reached ranged between 300-0 ng/mL and the control BC final dilution was 1:4. We tested in parallel our QC set of control and HD brain lysates, as well as a known HD BC sample. Although these are just Htt fragments, and not the full-length molecule, it indicates the relative effect of the PolyQ length on the resulting HTRF signal ratios, as shown in Fig. 7.

**Figure:**
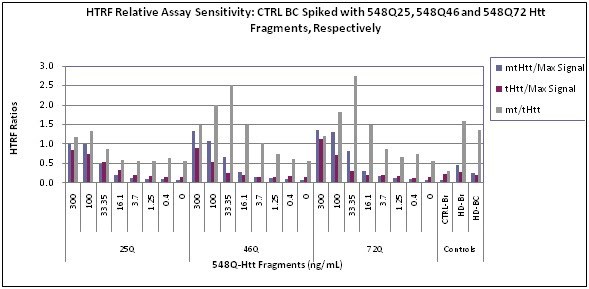
**Fig. 7: Relative Assay Sensitivity **Spike of 548Q25, 548Q46 and 548Q72 into control BC lysates. Spiking of the Htt fragments yielded 300-0 ng/mL 548Qn final concentrations and a 1:4 final dilution of the control (CTRL) BC. QC CTRL and HD brain lysates were used as described and an HD BC was used as a positive BC CTRL. The samples were tested in duplicates and the procedure as described in Material and Methods. HTRF ratios from a representative experiment were plotted as a function of 548Qn and their concentration: 300-0 ng/mL.

As a result of spiking of the 548Qn fragments into a matrix similar to the clinical matrix, we observed that the dynamic range for _mt_Htt (lack of nonspecific signal in 548Q25) is within<100-16.1 ng/mL for 548Q46 and<100-16.1 ng/mL for 548Q72. The dynamic range for _t_Htt is 100-3.7 ng/mL for 548Q25, and 300-16.1 ng/mL for 548Q46 and 548Q72, a PolyQ dependent shift to higher concentrations. However, the dynamic range for _mt/t_Htt, as shown in Fig. 7is 33.4-16.1 ng/mL (the lower limit is imposed by the lower limits of quantitation for both _mt_Htt and _t_Htt), which is a narrow range. 

**Intra-Assay Variability**: Fig. 8 is a representative example of the three assays performed for the assay qualification, demonstrating the reproducibility of the triplicate wells measured in the assay. The raw HTRF signals have a maximal Coefficient of Variation (CV) of <7%, which is low for a cell-based assay.

**Figure:**
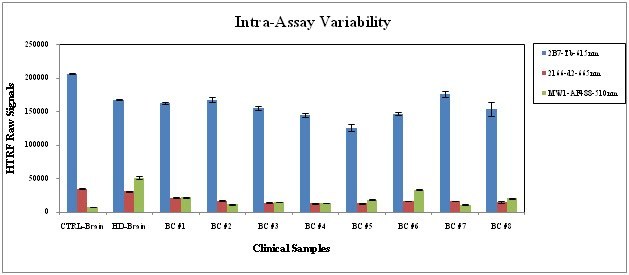
**Fig. 8: Assay Qualification: Intra-assay Variability **The raw HTRF signals from a representative assay (one of the three used for the assay qualification) evaluating 8 clinical BC and brain lysates from control and HD subjects. The HTRF Raw Signal values represent the averages (AVE) of triplicates ± standard deviation (STDEV).

**
 
**


**The Inter-Assay Variability**: Fig. 9 illustrates the average of MW1/2B7, 2166/2B7 and MW1/2166 ratios from three experiments. The STDEV for the HD samples is higher than that for controls, and its CV < 28%, which is satisfactory for a cell-based assay. The Z’ for _mt/t_Htt (MW1-AF488/2166-d2) calculated from the corresponding values of two sets of control and HD brain lysates/plate and including each of the three experiments was Z’=0.6. This Z’, is therefore based on a total of six determinations/QC sample/plate/experiment x 3 experiments = a total of 18 replicates/QC sample, which is very satisfactory. The results from the intra and inter-assay variability analyses indicate high technical reproducibility.

**Figure:**
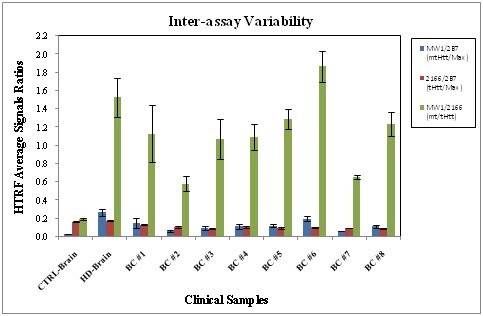
**Fig. 9: Assay qualification: Inter-assay Variability **The averages (AVE) of triplicates HTRF signal ratios ± standard deviation (STDEV) of 8 BC samples and the QC (control-CTRL and HD) brain lysates run are shown. The three experiments were run from replicate sets divided from the same samples and were carried out by two operators over the course of a week, using replicate aliquots of the same reagents. The assays were run as described in the Material and Methods.

**Biological Reproducibility**: We examined the biological reproducibility of the assay by measuring Htt in blood samples collected twice within a few weeks from the same individuals:

**Figure:**
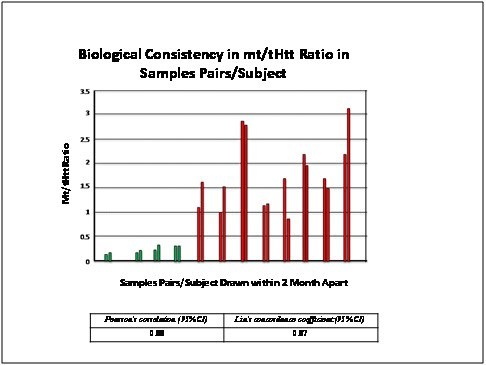
**Fig. 10: Assay qualification: Biological Consistency** 24 BC samples belonging to 12 (4 controls-CTRLS and 8 HD) subjects: 2 samples drawn per subject within 2 months apart were tested as described in Material and Methods. The samples are grouped as pairs and the corresponding _mt/t_Htt HTRF ratios are shown.

The results shown in Fig. 10 indicate excellent biologic consistency among the samples of the pairs tested, as confirmed by Pearson’s coefficient=0.88 and Lin’s concordance coefficient =0.87.

Following the assay qualification, we proceeded to write and issue a GLP compliant SOP. Using this SOP, we analyzed BC samples from 8 negative controls and 30 HD subjects (at different disease stages) in parallel to QC brain samples to demonstrate that this assay can differentiate between HD and controls based on the _mt/t_Htt levels.

**Figure:**
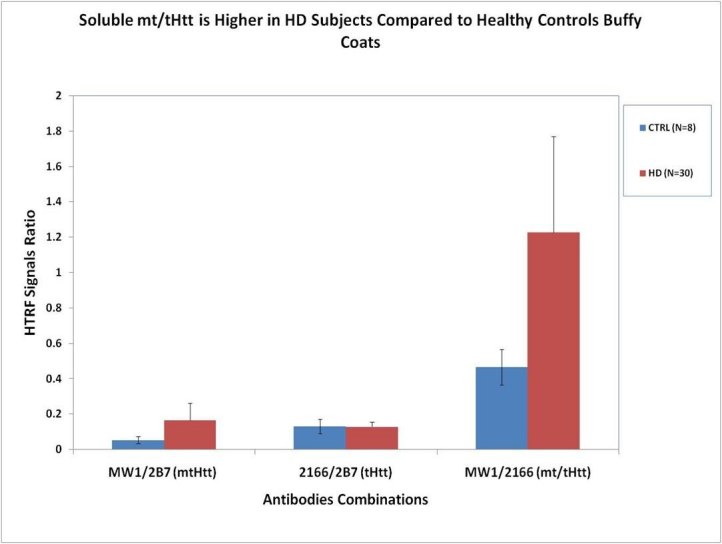

**Fig. 11: Soluble **_**mt/t**_ **Htt Detection in HD and Control Buffy Coat Samples **8 control (CTRL) and 30 HD subjects (in different disease stages) BC samples from one clinical site and QC brain lysates were tested according to the SOP described (Material and Methods), in parallel to QC CTRL and HD brain lysates (not shown). Median ± standard deviation (STDEV) values of the control (CTRL) and the HD groups represented as ratios: _mt_Htt, _t_Htt and _mt/t_Htt are shown.

**Measuring Clinical Buffy Coat Samples using SOP**

: Using the GLP-SOP, we were able to confirm our original findings (Weiss et al [4]) (Fig. 11) that the median _mt/t_Htt signals in BC samples from 30 HD subjects are higher than those measured in 8 control subjects. It is noteworthy that the samples tested were drawn from subjects at a single clinical site, ensuring minimal technical processing variability. The high standard deviation in the HD group reflects heterogeneity within this group which we hope to understand further by examining larger sample sets and clinical and laboratory covariates. 

**Discussion**

Due to the importance of soluble _mt_Htt protein and its cleavage products in HD as both the causative agent and as a therapeutic target, its measurement in peripheral biological samples has been long sought. While conventional immunoassays, including ELISA, have been unable to reliably measure soluble Htt in biological samples, HTRF is able to detect it in peripheral blood samples, including cell lysates [5]. Furthermore, the multiplexing approach[4] enables simultaneous detection of several epitopes on the soluble Htt molecule, such that we can measure both an expanded polyglutamine epitope (_mt_Htt) and an epitope distant from the polyglutamine tract (_t_Htt) (Fig. 1) in human tissues. Weiss et al [5] have shown that this HTRF method is able to specifically distinguish HD from control samples. Since soluble Htt levels would be an essential marker for measuring the biological effects of therapeutics targeting Htt, we optimized the assay to comply with GLP requirements, paving the way for its use in clinical samples for its validation as a biomarker for HD [8].

The optimization process we describe and quality control was enabled by the use of postmortem brain lysate samples from HD and control subjects. The selection criterion for optimal conditions for each variable was the highest ratio of HD/control when analyzing HTRF signal ratios of _mt_Htt and _mt/t_Htt. Based on this selection criterion we identified the 384 white plates with 15 µL/well as best suited for providing the strongest signal and enabling high throughput and the use of small sample volumes -15 µL (Fig. 2) with satisfactory intra-assay reproducibility. We determined that the reaction media needs to include the higher 200 mM KF, compared to 83 mM KF (Table 1) and that the reaction peaks between 2-3 hrs, while at 4^0^C (Fig. 3). We were able to identify Victor5X as a satisfactory plate reader for this assay, yielding optimal conditions with the following filters: excitation at 340 nm and emission at 665 nm, 615 nm and 510 nm (not shown).  We proceeded with the assay qualification using clinical BC samples from HD and control samples complying with GLP standards. We determined that the optimized assay is specific in detecting _mt_Htt and higher _mt/t_Htt levels in HD compared to control samples (Fig. 4), sensitive in detecting at least 80% of HD’ soluble _mt_Htt spiked into control brain lysates (Fig. 6), and linear within the dynamic range identified in each sample by analyzing two fold dilutions (Fig. 5). The tendency of the Htt fragments to aggregate hampered the efforts to make this assay fully quantitative using Htt protein standards, however we attempted to obtain a relative quantitative sensitivity measurement for _mt_Htt that is normalized to the signal that estimates the presence of _t_Htt, thus _mt/t_Htt signals, using purified 548Qn Htt fragments (Weiss et al [5] [4]) spiked into control BC samples (Fig. 7).The results using these Htt fragments indicate a narrow dynamic range for detecting _mt/t_Htt: 33.4-16.1 ng/mL for both the 548Q46 and 548Q72 Htt fragments. While it is impossible to know the absolute ranges we are indeed detecting by HTRF, serially diluting our BC samples, as we routinely do per our SOP, should enable us to capture the HTRF signals in spite of the narrow dynamic range. Based on the results presented with the spiked purified 548Qn Htt fragments, we are able to detect >3.7 ng/mL _mt_Htt and >16.1 ng/mL _t_Htt. These technical limitations could be circumvented only by purifying and spiking full-length soluble Htt into BC, thus finally determining the absolute quantitative soluble Htt measurements.

The measured HTRF ratios are compared between quality control brain lysate sets used at two ends of each plate with a Z’ ≥0.5 to ensure the quality of the analyses results. Analyses with this ratiometric approach offer the potential for informative use the HTRF assay in repeated measurements within subjects to evaluate the effects of disease progression or the effects of a potential therapy targeting Htt. To validate the measurement of soluble Htt as a clinical biomarker, we qualified the assay according to accepted GLP practices and found it to be technically reproducible with minimal (<7%) intra-assay (Fig. 8) and satisfactory (<28%, Fig. 9) inter-assay variability. The biological reproducibility between pairs of samples collected from subjects within a few weeks resulted in a coefficient of concordance >0.87 (Fig. 10), which is very promising for enabling longitudinal analyses. The results of our assay optimization led us to create a GLP compliant SOP, which we used to analyze clinical BC samples. The analysis of 30 HD samples in different disease stages and 8 control samples resulted in a specific discrimination of the two groups in regard to the _mt_Htt and the _mt/t_Htt ratios (Fig. 11). One possibility for the high standard deviation of _mt_Htt and the _mt/t_Htt ratios within the HD group is the heterogeneity of the subjects providing samples, who were selected without regard to disease severity or other clinical variables that could affect Htt levels. Variation in CAG length amongst the human samples could also play a role in variability between subjects, however since almost 90% of the expanded alleles from HD subjects are encompassed by a CAG repeat range of 40-48 (data from COHORT study, personal communication, Ray Dorsey), we don’t think that variability introduced by CAGn is likely to hamper the assay’s use as a biomarker. We plan to utilize sample sets from observational and therapeutic studies involving extensively characterized HD subjects to examine whether HTRF results are modulated by covariates such as age, gender, CAGn, disease stage, therapeutics and other biomarkers. 

**Acknowledgements:**

Susan Maya, MGH, Keith Malarick, MGH, Caleb Dresser, MGH, Aleksey Kazantzev, PhD, MGH, Luisa Quinti PhD, MGH, David Oakes, PhD, University of Rochester, Mary McCarthy, Perkin Elmer, Greg Warner, PhD, Perkin Elmer, Alan Lopatin, Anna Sinsigalli, Cisbio, Diane Bowers, Cisbio, Christopher Beck,University of Rochester.

**Funding Information:**

Funding for this work was provided by NIH grants: NINDS P01NS58793 and U01NS071789 and by the Novartis Institute for Biomedical Research.

**Competing interests:**

The authors have declared that no financial interests exist.The 2B7-Tb antibody has been made available to us by Novartis for purchase through Cisbio.

**Correspondence:**

Correspondence should be addressed to Steven M. Hersch: Hersch@helix.mgh.harvard.edu
